# Microfluidic Device for Patient-Centric Multiplexed Assays with Readout in Centralized Laboratories

**DOI:** 10.1021/acs.analchem.2c04318

**Published:** 2022-12-22

**Authors:** Janosch Hauser, Matilda Dale, Olof Beck, Jochen M. Schwenk, Göran Stemme, Claudia Fredolini, Niclas Roxhed

**Affiliations:** † KTH Royal Institute of Technology, Micro and Nanosystems, 10044 Stockholm, Sweden; ‡ KTH Royal Institute of Technology, Affinity Proteomics, Science for Life Laboratory, 17165 Solna, Sweden; § Karolinska Institutet, Clinical Neuroscience, 17177 Stockholm, Sweden; ∥ MedTechLabs, BioClinicum, Karolinska University Hospital, 17164 Solna, Sweden

## Abstract

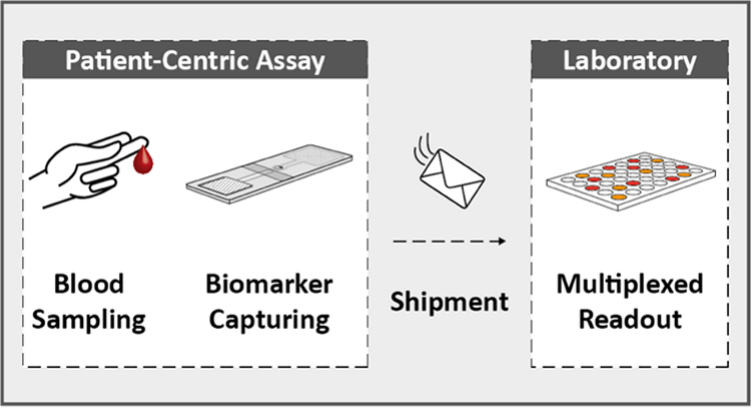

Patient-centric sampling strategies, where the patient performs self-sampling and ships the sample to a centralized laboratory for readout, are on the verge of widespread adaptation. However, the key to a successful patient-centric workflow is user-friendliness, with few noncritical user interactions, and simple, ideally biohazard-free shipment. Here, we present a capillary-driven microfluidic device designed to perform the critical biomarker capturing step of a multiplexed immunoassay at the time of sample collection. On-chip sample drying enables biohazard-free shipment and allows us to make use of advanced analytics of specialized laboratories that offer the needed analytical sensitivity, reliability, and affordability. Using C-Reactive Protein, MCP1, S100B, IGFBP1, and IL6 as model blood biomarkers, we demonstrate the multiplexing capability and applicability of the device to a patient-centric workflow. The presented quantification of a biomarker panel opens up new possibilities for e-doctor and e-health applications.

## Introduction

The COVID-19 pandemic led to a widespread adaptation of patient-centric sampling strategies where samples are taken in a home setting and shipped to a centralized laboratory for quality-secured analysis.^1,2^ Such patient-centric sampling offers obvious benefits, particularly when healthcare resources are sparse or travel should be avoided. In this development toward more patient-centric medicine, microfluidic devices can play an important role, e.g., by providing consistent sample collection and sample preparation on-chip. Especially immunoassays, when performed at the point of care (POC), could enable timely testing when a low turnaround time of analysis is highly needed, e.g., in first-aid settings (ambulance, physician offices, pharmacies). POC immunoassays also have great potential to assess time-critical and labile biomarkers, which are prone to degradation after sample collection.^3,4^ Various lab-on-chip-type immunoassays, e.g., based on centrifugal^5−9^ or paper-based^10−14^ microfluidics, were proposed to realize POC applications. In these approaches, analyte capturing is typically achieved using immobilized antibodies, e.g., in porous matrices^15^ or on bead surfaces.^16^ Accurate and comprehensive medical diagnostics often require assessments of multiple biomarkers, which can be achieved by multiplexing, the simultaneous detection of multiple analytes.^17^

However, without highly specialized readout equipment, it is difficult to match the sensitivity, reliability, and affordability offered by routine analyses in centralized laboratories.^18^ In addition, modern antibody-based proteomics technologies can simultaneously quantify thousands of proteins and detect concentrations down to fg/mL.^19^ State-of-the-art methods and instruments are designed to perform multiplexed biomarker capturing in solution (e.g., proximity extension assay (PEA)), on planar surfaces (e.g., electrochemiluminescence multiarray), or on bead surfaces (e.g., multianalyte profiling (xMAP)).^19^ While, for example, xMAP offers a high platform flexibility and the possibility to measure different analytes (protein, antibody, DNA, and RNA), the complexity of the instrumentation required for readout would not permit an on-site application from nontrained patients.^17^ However, such powerful multiplex readout technologies could open up new opportunities for patient-centric testing of biofluids, where the samples are collected in a home setting and shipped to a laboratory for quality-assured and multiplexed readout.

Good clinical practice relies on high-quality biological samples. Protein blood biomarkers are prone to degradation during the preanalytical phase between venipuncture and the biomarker measurement, which involves blood sample preprocessing (separation of serum or plasma^21−23^) and shipment to a laboratory for analysis. Inappropriate storage conditions and poor temperature control are the main factors responsible for laboratory testing errors.

Dried blood spot (DBS) sampling is a patient-centric sampling method, used to collect, ship, and store blood, that has the potential to alleviate some of the problems associated with the preanalytical phase. However, the capability to accurately quantify low-abundant biomarkers relies on the quality of the dried sample, the ability to quantify from it (through volume or intrinsic markers in the sample),^24^ the efficiency of recovery upon elution, and the sensitivity of the assay downstream since the elution procedure may lead to a dilution of 5–10 times or even more.^25,26^ Despite a number of issues related to DBS sampling, such as the impact of hematocrit on accurate quantification,^27,28^ devices for accurate volumetric collection^29^ have proven to overcome most issues associated with conventional DBS collection.^30−32^

Inspired by patient-centric sampling strategies, we present a capillary-driven microfluidic device designed to perform the first steps of a multiplexed immunoassay in a remote setting, enabling use of xMAP technology.^17^ The device contains dry magnetic beads, conjugated with antibodies, for protein capturing, which allows us to directly isolate target analytes at the time of sample collection. This direct target isolation step allows circumventing an unwanted sample dilution, required for other patient-centric sampling strategies.^20^ Further, drying the antibody–protein complexes enables simple shipment and allows us to make use of the advantages of the xMAP technology implemented in highly specialized laboratories.

## Device Design and Envisioned Workflow

**Figure 1:**
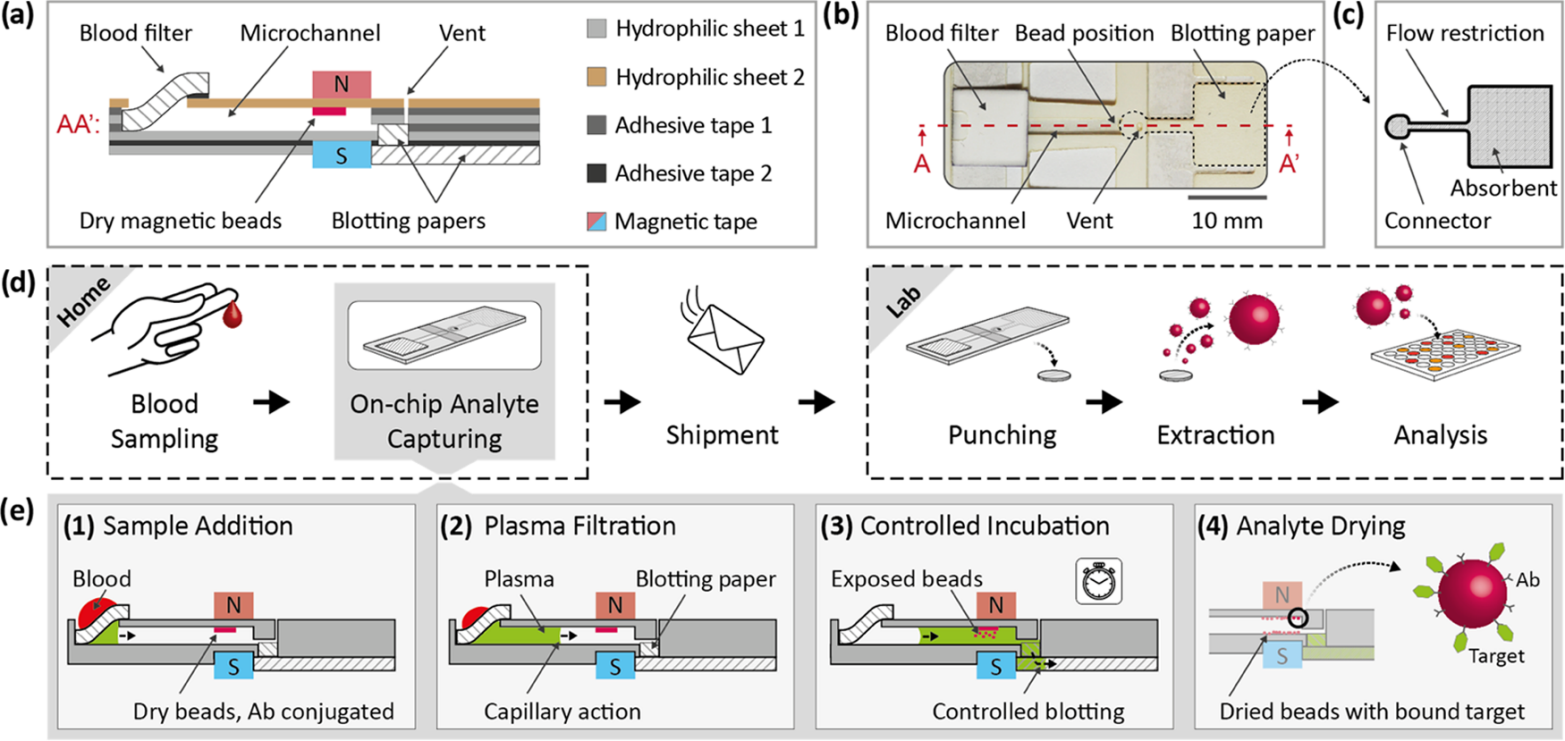
(a) Schematic cross-sectional view of the device (along A–A’), indicating the laminated materials. (b) Top view picture of the microfluidic device with dimensions of 15 × 40 mm^2^. (c) Geometry of the blotting paper, providing a controlled volumetric flow rate. (d) Patient-centric workflow for the bead-based assay starts with blood sampling and on-chip analyte capturing in a home setting, followed by dry shipment and finally analysis in a centralized laboratory. (e) On-chip analyte capturing consists of four steps: (1) sample addition, (2) plasma filtration, (3) time-controlled sample incubation, and (4) analyte drying before shipment.

The device is designed to enable a patient-centric workflow for timely quantification of labile critical biomarkers (e.g., frequent monitoring of the elderly at home; patients reaching rural and/or hospital emergency rooms), where optimally the device should return to a central laboratory for analysis within 24 h. For capillary-driven blood plasma filtration in remote settings, the device has a blood filter in conjunction with a hydrophilic microchannel designed to meter a predefined volume of plasma from an unknown volume of whole blood (Figure 1a), as previously shown by us.^33,34^ To allow simple shipment of the device to patients, the microfluidic device contains dry magnetic beads conjugated with antibodies for target protein capturing. These magnetic beads can be conveniently extracted from the device, which enables direct interfacing with existing downstream routine analysis in the laboratory. The device also contains magnetic tapes, to collect the magnetic beads, and blotting paper, to absorb the sample liquid. A vent above the blotting unit allows air to escape. Figure 1b shows a photograph of the device, and Figure 1c indicates the geometry of the blotting paper, with a connector piece in contact with the microchannel, a flow restriction, and an absorbent acting as a capillary pump. The geometrical flow restriction regulates the volumetric flow rate.^35^

Figure 1d shows the envisioned assay workflow. First, in a home setting, the patient takes a blood sample from the fingertip and adds it to the microfluidic device, which starts the on-chip biomarker capturing. Drying the captured target biomarker allows a simple and biohazard-free transfer of the device to a centralized laboratory where the remaining assay steps are carried out. Figure 1e shows the on-chip biomarker isolation steps. The assay is started by adding blood (Step 1) to the blood filter, which provides blood plasma (Step 2). Blood plasma filling the microfluidic channel by capillary action reaches the dry beads and the blotting paper, which starts the incubation time. The time the beads are exposed to the target protein is controlled by the blood plasma volume in the microchannel and the volumetric flow rate, which is defined by the blotting paper (Step 3). The volume of the microchannel is designed to contain 10 μL. We previously demonstrated on-chip blood plasma volume metering^34^ which is an essential feature of the design concept. In the present study, however, most experiments are done with a metered plasma volume that is manually added to the device. The incubation time stops when all plasma is drained and the beads are no longer exposed to the target protein. The device, containing beads (now carrying bound target proteins), is then left to dry under ambient conditions and shipped to a laboratory at room temperature for analysis (Step 4). In the laboratory, the part of the device containing the beads is punched out for bead extraction and subsequent analysis in a well plate (Figure 1d).

## Experimental Section

### Device and Assay Materials

As indicated in Figure 1a, the device consists of four layers of hydrophilic sheets [hydrophilic sheet 1 (3R3028 Type C, Xerox) and hydrophilic sheet 2 (3R98202, Xerox)], three layers of double-sided adhesives [adhesive tape 1 (62571, Tesa, Germany) and adhesive tape 2 (8132LE, 3M, Digi-Key)], blotting paper [Ahlstrom grade 601 (Ahlstrom Filtration LLC)], a blood filter (SG regular, IPOC, Canada), and magnetic tapes (Nd 10 mm, Supermagnete, Germany). The device contains magnetic beads (MagPlex magnetic microspheres, Luminex) conjugated with antibodies, as described previously.^36,37^ As a negative control, we coupled mouse IgG (mIgG, PMP01, Lot-031114, Bio-Rad, Sweden) to the beads. The implemented assay kits (DuoSet, R&D Systems) were human C-Reactive Protein/CRP (DY1707, Lot-P248178), human IL6 (DY206, Lot-P253525), human MCP1 (DY279, Lot-P294761), human IGFBP1 (DY871, Lot-P270852), and human S100B (DY1820, Lot-P277429). All of the assay kits listed above included the respective recombinant standard protein, capture, and detection antibodies, while the reagent diluent was taken from the ancillary reagent kit (DuoSet DY008, R&D Systems). Phosphate-Buffered Saline, 0.05% Tween 20 (PBST) was prepared by mixing phosphate-buffered saline (PBS) (#09-9400, Medicago, Sweden) and Tween 20 (BP337-500 Fisher BioReagents, Thermo Fisher, Sweden). Assays were carried out in 96-well plates (GREI675101, Greiner Bio-One, BioNordica, Sweden). Human whole blood samples were obtained from a blood collection center (Blodcentralen, Stockholm, Sweden) in BD Vacutainer EDTA tubes. Ethical permission for the study was obtained from the regional ethical board (EPN Stockholm, Dnr. 2015/867-31/1). To obtain plasma samples, we centrifuged the blood samples at 500 g for 5 min. Additionally, we used pooled human plasma (#HMPLEDTA2 Human K2 EDTA mixed gender plasma pool, Seralab, U.K.).

### Device Fabrication

The device was fabricated using lamination technology, as described previously.^33,34,38^ Individual layers of hydrophilic sheets, adhesive tapes, magnetic tapes, and paper were structured using a laser cutter (VLS 2.30, Universal Laser Systems, Austria). Blood filters were cut into 10 × 10 mm^2^ pieces using a scalpel. The edges of the blood filters were impregnated with liquefied wax to prevent blood cell leakage, as described earlier.^33^ The layers were aligned using two alignment pins and laminated at room temperature using a laminator (Heat Seal Pro H600, GBC). The dimensions of the microchannel are 2 × 15 × 0.26 mm^3^. Before closing the device with the last layer, 2 μL of bead suspension, containing approximately 400 beads per analyte of interest, was added to the hydrophilic sheet 2 and dried in the dark overnight at room temperature. The beads were positioned to be near the connector piece of the blotting paper. This ensures that the plasma front reaches the beads and the blotting paper almost simultaneously, which allows for a controlled incubation time. Hydrophilic sheet 2 was chosen because it has a higher contact angle with water (75°) than hydrophilic sheet 1 (10°), resulting in a smaller area with beads inside the microfluidic chamber. A small area with beads is beneficial for consistent incubation time, as individual beads are exposed to liquid for a similar time.

### Bead Extraction and Readout

For bead extraction from the devices, the magnetic tape was removed. The part of the microchannel containing the beads (6 ×10 mm^2^) was cut out and transferred to Eppendorf tubes containing 100 μL PBST. The microchannel part was oriented so that one of its openings was facing the bottom of the tube, allowing PBST to enter by capillary forces. The beads, now in contact with PBST, were eluted by three alternating short vortexing (2 s) and centrifugation steps (2 s). Vortexing was performed on the highest setting of a vortex mixer (Vortex-Genie 2, Scientific Industries). Centrifugation was done on a mini benchtop centrifuge (Mini Star, VWR, Sweden) at 6000 rpm. After a final vortexing step, we transferred the liquid, now containing the beads, from the tubes to a well plate.

The remaining assay steps were carried out according to a standardized in-plate protocol. In short, 50 μL of biotinylated antibody diluted in reagent diluent (according to manufacturer instructions for each assay) was added to the wells in the plate and incubated for 2 h at room temperature, in the dark, on a shaker at 650 rpm. After incubation, the plate was spun down and washed three times with 100 μL of PBST. Beads were then incubated with 50 μL streptavidin phycoerythrin (SAPE) for 20 min at room temperature. Finally, the plate was washed three times with 100 μL of PBST.

Fluorescent readout was obtained on a FlexMAP 3D (Luminex) instrument. The software xPONENT (Luminex) provides median fluorescence intensity (MFI) values for relative quantification. Curve fitting and extrapolation of concentrations were performed with Belysa Immunoassay Curve Fitting software (Millipore) and R programming environment. Standard curves were generated using a five-parameter logistic (5PL) curve fit.

The number of beads extracted for readout was sufficient throughout the study, with typically >100 beads per bead ID. Luminex bead-based assays follow the principle of the ambient analyte theory as described by Roger Ekins.^39^ The amount of capture antibody in the sandwich immunoassay setup is decreased from a macrospot (e.g., an ELISA where the antibody is coated on the well of a microtiter plate) to a microspot (bead) where only a small fraction of the present target analytes is captured, proportional to the analyte concentration in solution. According to the Luminex manufacturer’s instruction, a number of beads between 25 and 50 is enough to produce a statistically accurate result. The minimum number of events/region was therefore set at 100.

### Five-Parameter Logistic (5PL) Curve Fit

**Figure 2:**
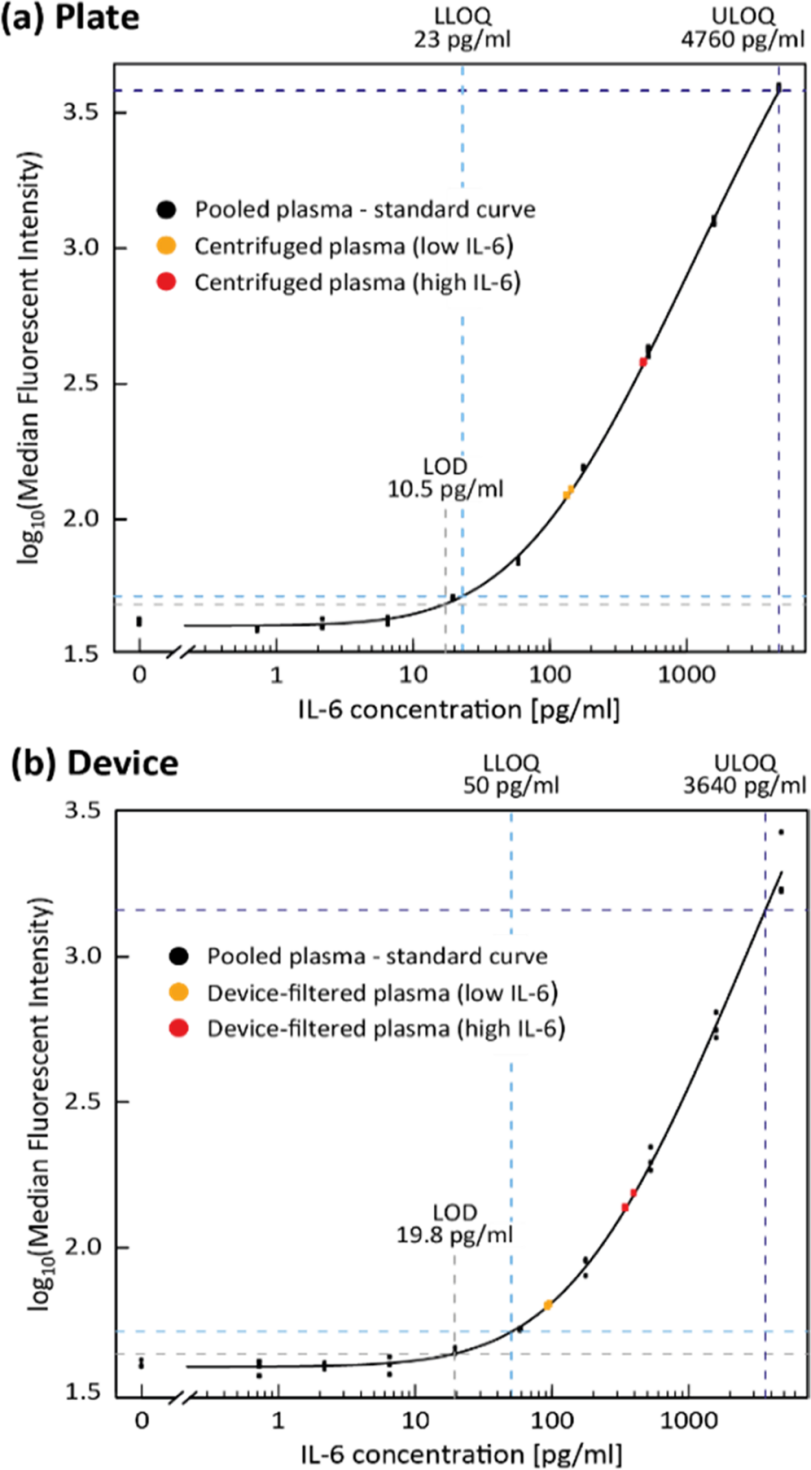
Calibration curves obtained using spiked pooled plasma in (a) plate and (b) devices. Limit of detection (LOD), lower limit of quantification (LLOQ), and upper limit of quantification (ULOQ) were calculated and are indicated in the plot. The concentration of unknown samples is obtained from the standard curve by projecting median fluorescent intensity readings onto the respective calibration curve and extrapolating the corresponding concentration value.

To obtain standard curves for the device- and plate-incubated beads, we used spiked pooled plasma (black data points in Figure 2). A five-parameter logistic (5PL) curve fit resulted in the respective standard curves and allowed us to compute the limit of detection (LOD), lower limit of quantification (LLOQ), and upper limit of quantification (ULOQ). The dynamic range, between LLOQ and ULOQ, resulted in 23–4760 and 50–3640 pg/mL for plate- and device-incubated beads, respectively. LOD, LLOQ, and ULOQ are calculated from median fluorescent intensity (MFI) as followed:

LOD = mean MFI(blank) + 3 standard deviations (blank).

LLOQ = mean MFI (blank) + 10 standard deviation(blank).

ULOQ = mean MFI (highest calibrator) – standard deviation (highest calibrator).To obtain the IL-6 concentration of the spiked whole blood samples, we projected MFI results onto the respective calibration curve (plate, device) and extracted the corresponding concentration values (Figure 2).

### Bead Drying before and after Sample Incubation

The effect of bead drying before and after sample incubation was tested in plate. Here, we assumed that the shipping step from healthcare provider to patients could be achieved in a week and that from patients to the laboratory could be achieved within 3 days. Therefore, we tested the stability of beads covalently coupled to antibodies up to 7 days before being incubated with a sample and of beads carrying an antibody–protein complex for up to 3 days. In both experiments, a dilution of recombinant CRP standard was used as sample. A twofold, eight-point dilution series starting at 4 ng/mL was prepared in triplicate. CRP recombinant standard protein and the commercial dilution buffer (reagent diluent) were included in the DuoSet kit (DY008, R&D Systems). Beads were washed from storage buffer (when Drying Before) or from diluted CRP (when Drying After) with PBST. Then, after any residual liquid was removed, the plate was covered with a lid and allowed to dry in a plastic bag containing silica beads (desiccant) at room temperature.

### Device-Controlled Incubation Time

To understand the incubation time dependency of the assay, we performed a CRP assay in plate with different incubation times (2, 5, 10, and 120 min). CRP standards (triplicate) were used as samples in a twofold dilution series with eight points, starting from 4 ng/mL.

In the microfluidic device, the incubation time is controlled by the sample volume and the volumetric flow rate. To study the influence of the blotting paper on the volumetric flow rate, we fabricated devices with different geometries of the flow restriction (Figure 1c) (width 1 mm: length 2 mm, 4 mm; width 2 mm: length 2, 4, 8 mm). Per geometry, we fabricated six devices without a blood filter and tested them by adding 10 μL of blood plasma from one donor. For comparison, we fabricated six devices (flow restriction width: 1 mm; length: 8 mm) and used the devices with 10 μL of reagent diluent. To assess the device-controlled incubation time, we recorded videos of the microfluidic sequence and calculated the time between the first wetting of the bead area inside the microchannel and the moment when all liquid was removed from the bead position.

### On-Chip Assay: Device versus Plate

To test the on-chip assay, we prepared 48 devices containing dry beads. We added 10 μL of sample to each device. As a reference, we prepared an assay in plate with 10 min of incubation. CRP standards (triplicate) were used as samples in a fourfold dilution series with eight points, starting from 50 ng/mL. We used devices without blood filters to focus on the on-chip time control. For 24 devices, the incubation time was controlled manually by filling the microchannel and blotting the liquid after 10 min. The remaining 24 devices had an integrated time control with a blotting paper. The flow restriction was 1 mm wide and 8 mm long, corresponding to approximately 10 min of incubation time for reagent diluent which is less viscous than blood plasma.

### On-Chip Assay: Whole Blood and Plasma

To test the on-chip assay with whole blood and plasma samples, we prepared 36 devices, 6 with blood filters and 30 without. We chose IL6 as a biomarker because of its low endogenous levels in healthy adults, which allowed us to spike human blood and plasma with IL6. The devices with blood filters were used to assess the applicability to a patient-centric workflow, where on-chip plasma filtration is an enabling element. We used fresh whole blood from one donor with a hematocrit of 45% and spiked it to obtain three different IL6 concentrations: (i) blank (endogenous), (ii) low (measured 141 pg/mL); (iii) high (measured 494 pg/mL). Endogenous levels of IL6 were undetectable. For incubation in plate, we centrifuged the same blood samples at 500 g for 3 min to separate the plasma from the cellular blood fraction. The devices without blood filters were meant to study the effect of blood plasma as a sample matrix. We used pooled plasma with undetectable endogenous levels of IL6 to prepare a threefold serial dilution with nine points, starting from 4800 pg/mL, which allowed us to generate standard curves (plate, device) using a five-parameter logistic (5PL) curve fit. Using the respective standard curve (plate, device), we converted the MFI results from the whole blood samples into concentration values (Supporting Information). The flow restriction of the blotting paper was 1.5 mm wide and 6 mm long, corresponding to approximately 15 min of incubation time for plasma. For all samples, we avoided changing the viscosity by keeping the spike-to-sample volume ratio below 0.05.

### On-Chip Assay: Multiplexing

To test the multiplexing capacity of the on-chip biomarker capturing, we fabricated 24 devices without blood filters and included beads with five different bead IDs, coding for different target antigens, in each device. The bead IDs carried antibodies for CRP, IL6, MCP1, S100B, and IGFBP1. Two additional bead IDs were included as negative controls, one carrying antibodies for mouse IgG, and bare beads, not carrying antibodies. While the multiplexing capacity of the microfluidic device is based on the xMAP technology by Luminex, the device has key enabling elements, as introduced above, that enable us to exploit the advantages of xMAP in remote settings. As a sample, we used commercial reagent diluent spiked with 4 ng/mL of IGFBP1, 3 ng/mL of S100B, 4 ng/mL of CRP, 2 ng/mL of IL6, and 1 ng/mL of MCP1 and prepared a twofold dilution series with eight different concentrations. We added 10 μL of sample to each device and prepared triplicates for each of the eight concentration points. As a reference, we prepared an assay in plate with 15 min sample incubation. The flow restriction of the blotting paper was 1 mm wide and 8 mm long, corresponding to approximately 10 min of incubation time for the reagent diluent used in this experiment.

## Results and Discussion

### Bead Drying before and after Sample Incubation

**Figure 3:**
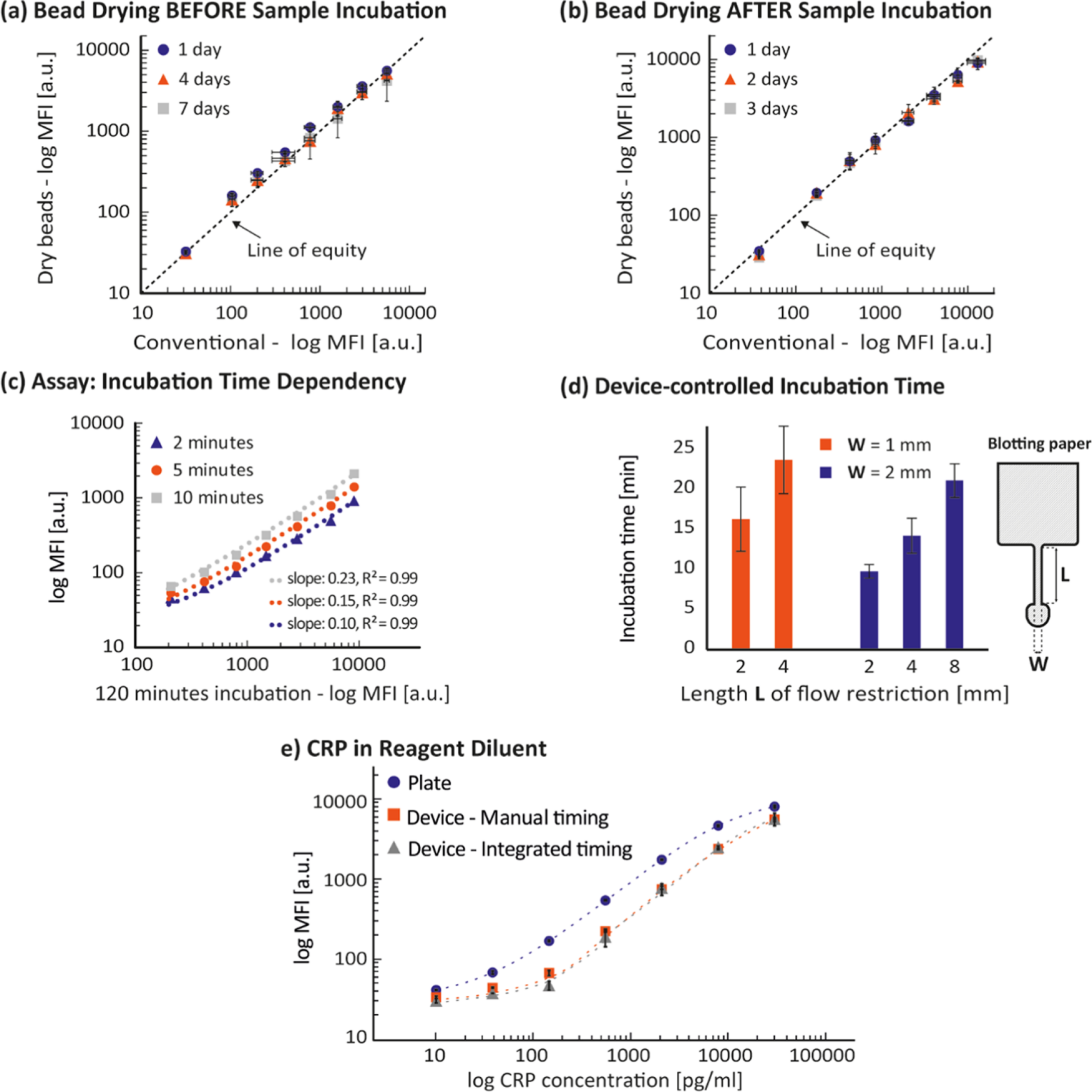
Median fluorescent intensity (MFI) from beads that were dried (a) 1, 4, and 7 days before and (b) 1, 2, and 3 days after sample incubation plotted against MFI from fresh beads that were not dried (conventional). The axes present the MFI data in log scale. The dashed and straight lines indicate the trendline and line of equity, respectively. (c) MFI of the CRP assay performed with different incubation times, relative to MFI values of the same assay with 120 min of sample incubation. The dotted lines indicate linear curve fits that are curved due to the log scales of the axes. (d) Incubation time, defined as the time the sample is in contact with the beads, for devices with different flow restrictions of the blotting paper. The bars and error bars represent mean values and standard deviations of six repetitions, respectively. (e) MFI signal over CRP concentration of assays with CRP in reagent diluent where the sample incubation was performed in a well plate, devices with manual time control, and devices with integrated time control.

Two drying steps are crucial for the patient-centric assay workflow. To allow storage and simple shipment to patients, the microfluidic device needs to contain dry magnetic beads, covalently conjugated with antibodies. For simple and biohazard-free shipment to a centralized laboratory, the beads carrying the antibody–target complexes have to be dried. Antibodies covalently coupled to beads are expected to maintain their biological activity, dehydration (e.g., by lyophilization) is indeed a common procedure adopted by the pharmaceutical and biotechnology industry to preserve and ship proteins regardless of cold chain transport availability. However, antibody–target complex stability upon shipment at room temperature could be a critical step. To test the stability upon drying of the antibodies coupled to magnetic beads (before sample incubation) and of the bead–antibody–target complex (after sample incubation), we used a CRP assay as a model. Figure 3a shows a comparison between a conventional CRP assay performed according to a validated SOP, which involves the use of freshly prepared beads stored at 4 °C in storage buffer and the same assay where the beads were dried for 1, 4, and 7 days before the sample incubation. The measured MFI of both assays plotted against each other exhibit a linear relationship (*R*^2^ = 0.99) with a good match between the linear curve fit and the line of equity. This shows that drying the bead–antibody complex before sample incubation does not negatively affect the CRP assay. Figure 3b shows the comparison between a conventional assay and the same assay where the beads were dried for 1, 2, and 3 days after the sample incubation. The MFI values of both assays plotted against each other exhibit a linear relationship (*R*^2^ = 0.99), where the linear curve fit and the line of equity are close to identical. This shows that drying the immune complexes after sample incubation does not negatively affect the CRP assay. Overall, the presented results show that both drying steps are feasible within 3 days and support the feasibility and applicability of the on-chip assay presented here for a patient-centric testing strategy. However, longer stability studies will be needed to define the maximum storage time.

### Device-Controlled Incubation Time

To assess the effect of different sample incubation times, we performed four assays in plate. Figure 3c shows the MFI signal of three assays that were performed with different sample incubation times (2, 5, 10 min) in relation to the MFI signal of the standard assay with 120 min of sample incubation (conventional). For all three incubation times, the results exhibit a linear relationship to the conventional assay with a coefficient of determination *R*^2^ = 0.99, indicating that the data sets have a strong linear correlation. The linear curve fits reveal slopes smaller than 1 for all three assays with increasing slopes for increasing sample incubation times. The strong linear correlation indicates that a sample incubation time as low as 2 min could be feasible. However, the limit of detection (LOD) is affected by low sample incubation times, increasing from 1.8 pg/mL (10 min) to 3.7 pg/mL (2 min). Therefore, long incubation times are favorable, with the limiting factor being the feasibility, reliability, and consistency of a device-controlled incubation time. Longer incubation time is also favorable as the relative contribution from time variability in the filtration event (e.g., due to blood hematocrit^33^) would be low.

To study the capability of the device to control such incubation times by volumetric flow rate, we fabricated devices with different geometries of the blotting unit. Figure 3d shows the device-controlled incubation time for various geometries of the blotting paper, using blood plasma as the sample. The incubation time increases both for increasing lengths and decreasing widths of the flow restriction. An in-depth characterization can be found in the literature.^35^ Here, the achieved device-controlled incubation times ranged between 9 and 24 min with the chosen geometries. The average CVs (coefficient of variation) of devices with a flow restriction width of 1 mm and 2 mm are 21 and 11%, respectively. This means that a wider flow restriction allows for more consistent timing. Individual CVs below 10% indicate that capillary means can be employed to consistently control the sample incubation time. The devices (*n* = 6) used with reagent diluent resulted in a device-controlled incubation time of 10.9 ± 0.9 min. Compared to plasma, reagent diluent was blotted faster, suggesting that the protein content and increased viscosity of plasma change the flow properties. Overall, the results show that assays could be performed at incubation times as low as 2 min and that the device can control such incubation times, up to 24 min.

### On-Chip Assay: Device versus Plate

To apply and assess the integrated incubation time control, we performed three CRP assays: in plate, in devices with manual timing, and in devices with integrated timing, all with approximately 10 min of incubation time. Figure 3e shows the result of the three assays. All curves exhibit linear dose–response curves in the linear range of 15.6–10,000 pg/mL. Data generated with manual and integrated time control show a strong positive relationship. A linear curve fit between the two device MFI values results in a slope of 1.02 and a coefficient of determination *R*^2^ = 0.99 and highlights the capability of the device to autonomously perform the sample incubation step for downstream readout in a high-throughput instrument.

### On-Chip Assay: Whole Blood and Plasma

The performance of the on-chip assay with human whole blood and plasma was evaluated with a spike-in experiment. IL6 was chosen as a model of a low-abundant biomarker. Since the assay performed in plate and in the device are technically different and to avoid the application of a correction factor, we decided to generate assay-specific standard curves. A calibration curve was prepared by diluting IL6 in human plasma from healthy donors with undetectable levels of IL6 in the range 0.7–4800 pg/mL. Aliquots of each calibrator were run in the plate (Figure 2a) and in the device in parallel (Figure 2b). The MFI values obtained were plotted against the respective spike-in concentrations, fitted using a Five-parameter Logistic (5PL) Curve Fit. LODs calculated for plate and device were, respectively, 10.5 and 19.8 pg/mL, and LOQs were, respectively, 23 and 50 pg/mL. The average CV between triplicate measurements of plasma samples in the device was 6%, (Supporting Information, Table S1) which highlights the consistency of the device. Despite the fact that the LOD and LOQ for the device are slightly higher compared to those obtained for the same calibration curve in plate, the assay’s sensitivity can still be considered satisfactory to meet the cut-off for determining high levels of IL6 in patients. For IL6, for example, a baseline value of <10 pg/mL has been estimated, which may rise over 30 pg/mL in bile duct cancer and gastric cancer; over 100 pg/mL in COVID-19; and up to 500 pg/mL in infants.^40−43^

**Figure 4:**
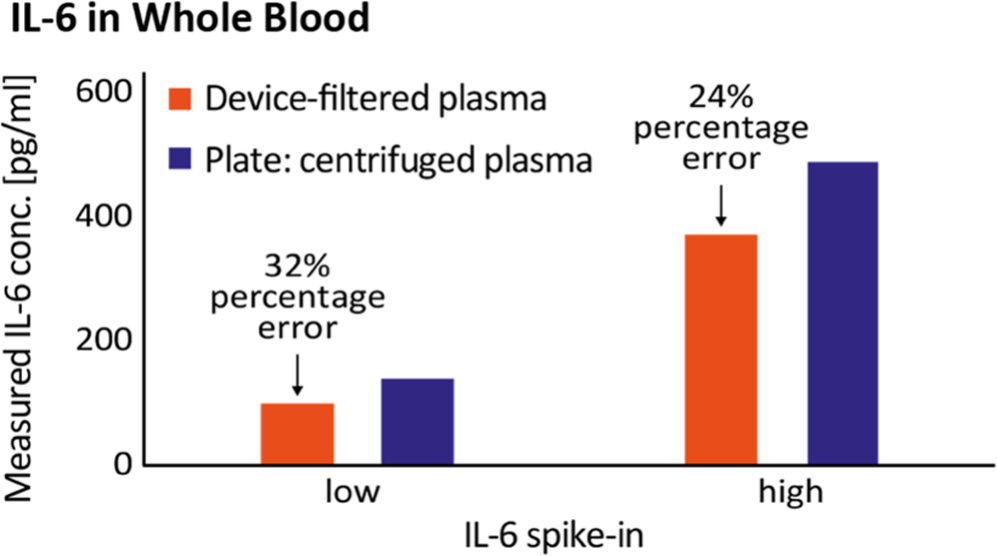
Comparison of device-filtered plasma and centrifuged plasma with sample incubation in device and plate, respectively. Measured IL-6 concentrations are obtained as detailed in the Experimental Section.

To evaluate the accuracy of the quantification obtained from the device with respect to the gold standard method (immunoassay in plate), we measured the IL6 concentration in two unknown plasma and blood samples, on device and in plate. Figure 4 shows the IL6 concentrations obtained from the measurement of a spiked whole blood sample. For the analysis in plate, an aliquot of the whole blood sample was centrifuged to obtain plasma. On the device, blood plasma was obtained by on-chip filtration. IL6 quantification was performed using the respective plasma standard curve generated on device and in plate (Figure 2). IL6 concentrations obtained in plate from centrifuged plasma were 141 pg/mL (low) and 494 pg/mL (high). For device-incubated beads with device-filtered plasma, measured concentrations were 96 pg/mL (low) and 377 pg/mL (high). Percentage errors for low and high concentrations measured in the device with respect to the assay in plate were 32 and 24%, respectively. This could be due to decreased protein levels in filtered plasma with respect to centrifuged plasma, most likely due to protein binding to the filter material, as shown previously.^33^ The 24 and 32% errors between the device and the well plate fall within a range of variability, which is considered acceptable for technical replicates. Moreover, the range of variability for IL6 has been estimated to be higher than 20% between healthy individuals and patients affected by high systemic inflammation. For example, concentration values reported in the literature for IL-6 in the blood varied between 0 and 43.5 pg/mL for healthy donors, to reach more than 10,000 pg/mL levels in adults with sepsis.^44^ Therefore, an error ≤ 30% could be considered satisfactory at this stage of device development. For a final product that aims to be implemented in a clinical setting, it will be essential to evaluate the effects of the blood filter and refine the filtration strategy to reduce protein losses for every assay and target biomarker. Overall, the results show that the device can handle blood plasma as a matrix and that on-chip blood plasma filtration can be a feasible approach to obtain plasma in settings where centrifuges are not readily available.

### On-Chip Assay: Multiplexing

**Figure 5:**
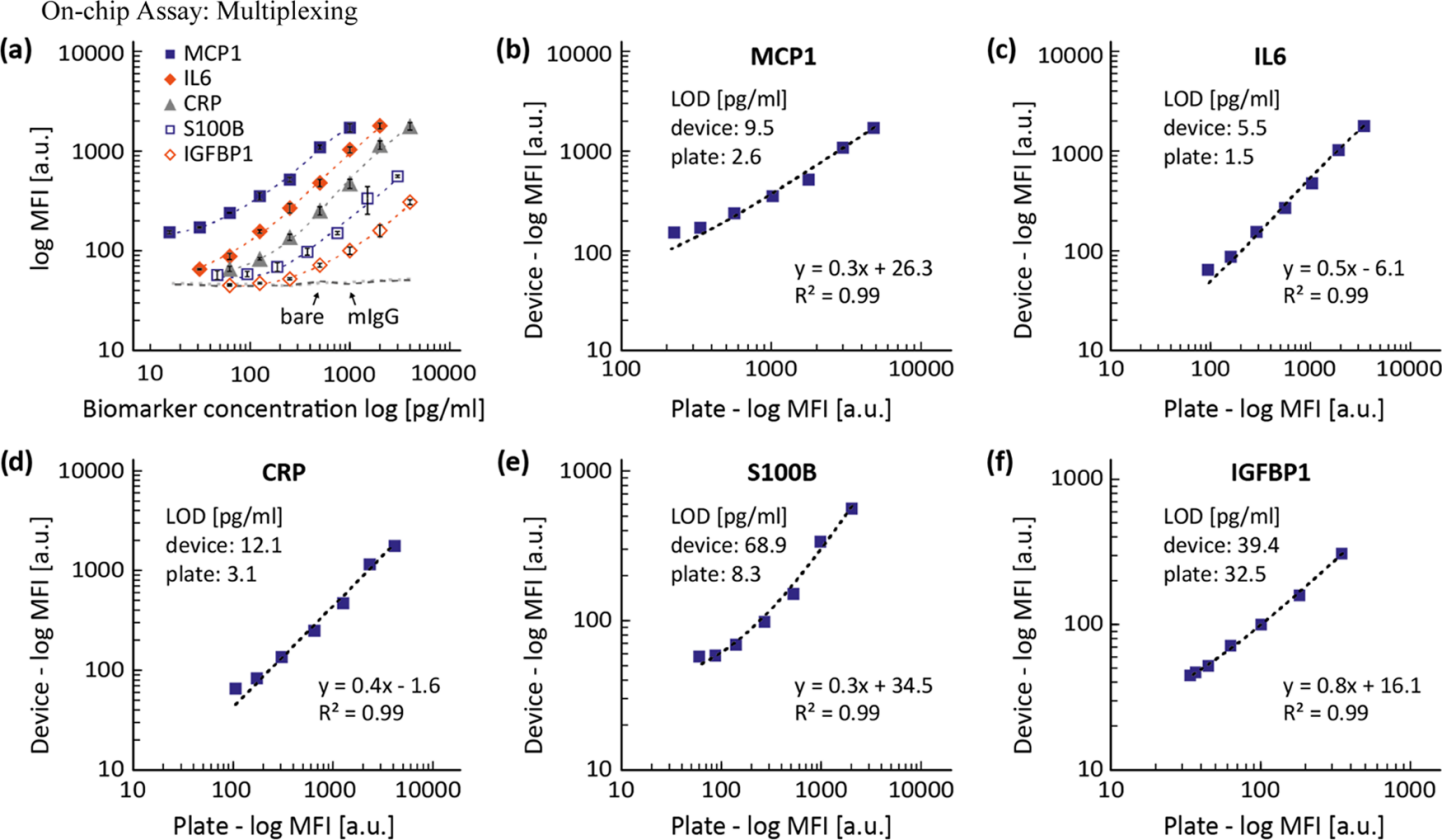
Multiplex quantification of five blood biomarkers using the microfluidic device. (a) MFI values for five biomarkers and negative controls (bare beads and mIgG) were plotted against the concentration of the standard proteins on axes in log scales. The data points represent average values from triplicate measurements and the error bars indicate standard deviations. MFI values obtained in the device plotted against intensity signals from plate for MCP1 (b), IL6 (c), CRP (d), S100B (e), and IGFBP1 (f). Data points are calculated average values from triplicate measurements. Dotted lines represent linear curve fits that are curved due to the log scales of the axes.

The multiplexing capacity of the microfluidic device was tested on a panel of five biomarkers used for diagnosing or monitoring traumatic brain injuries (S100B),^45^ rheumatoid arthritis (MCP1),^46^ gastrointestinal cancer (IFGBP1),^47^ and sepsis (CRP, IL6).^48^ Additionally, we measured MFI values from mIgG and bare beads as negative controls. Since most of the biomarkers selected were detectable in blood from healthy donors at relatively high levels, but we wished to test our system in a broader range of concentration, we decided, for this experiment to spike-in recombinant proteins in commercial reagent diluent (see the Experimental Section). Reagent diluent is a protein solution used to prepare the calibration curves according to the manufacturer’s instructions of the assays used here and resembles the matrix effect of a biological sample. Figure 5 shows the MFI values for the five biomarkers over biomarker concentration and the signal from the negative controls. Dilutional linearity, above LOD and below ULOQ, was confirmed for all biomarkers. The MFI values for the bare beads and mIgG indicate a low background level. The average CV for all of the analytes calculated on technical replicates was <6%, (for MFIs) and <9% (for concentration) indicating the consistency of the data generated with the device (Supporting Information, Table S1). Figure 5b–f shows MFI from device-incubated beads against plate-incubated beads for the different biomarkers. For all of the model biomarkers, a linear curve fit with coefficients of determination *R*^2^ > 0.98 indicates a good linear relationship between the plate- and device-incubated samples. Such linear relationships could be used as external standard curves to quantify biomarkers in samples obtained by a patient-centric workflow.

A very good concordance between MFI values in plate and device was observed for all of the targets but S100B [MFI shifted to a lower range in the device (Figure 5a)]. However, since MFI values are a semiquantitative unit and cannot be used as a measure of sensitivity, we calculated the LODs for each assay (Figure 5b–f). For all tested proteins, the assays performed in the plate achieved lower LODs.

The increase in LOD for the device ranges from 1.2-fold for IGFBP1 to 8-fold for S100B, with a 4-fold increase for all others showing to be assay dependent. The shorter incubation time and lack of active mixing could indeed affect differently the performance of each antibody pair. It is important to note that this aspect is independent of the device but represents an intrinsic feature that will need to be evaluated for each immunoassay implemented on the device. Despite the increase in LOD between the assay in plate and in the device, the LOD and LOQ obtained still meet the analytical performances needed to detect clinical levels of the biomarkers tested here, which have been estimated in recent literature to be CRP: <0.3 mg/dL;^49^ IL6: 0-43.5 pg/mL; MCP1: 190 μg/mL;^50^ IGFBP1: 60–440 ng/mL; S100B <0.105 ng/mL (all for healthy individuals).

Overall, these results indicate that the device can multiplex several analytes. Even though Luminex technology and other modern multiplexing platforms are currently mostly used in research laboratories, they open up unprecedented possibilities for next-generation biomarker assessments. Such multiplexed assays can enable the quantification of clinical biomarker panels to achieve higher diagnostic and prognostic accuracy.^51−53^

## Conclusions

We presented a microfluidic device that can perform the critical target binding step of a multiplexed immunoassay at the time of sample collection. The applicability to a patient-centric workflow results from three key features of the device. First, the device contains dry beads, conjugated with antibodies for protein capturing. We showed that drying the beads on the device does not negatively affect the performance of the readout. Second, the device has an integrated blood filter for on-chip blood plasma filtration. Third, the device does not require any external equipment, uses only capillary forces to move the liquids, and can control the sample incubation time without any user interaction. An average CV of 10% shows the consistency of the device-controlled incubation and the consistency in extracting beads carrying the captured biomarkers. A drying step after the sample incubation allows simple shipment from the patient to a laboratory. This allows us to make use of the multiplex capacity, analytical sensitivity, reliability, and affordability offered by routine analyses in centralized laboratories. Using seven different bead IDs, encoding for five known blood biomarkers and two negative controls, we demonstrated the multiplexing capability of the device. With blood plasma as a matrix and IL6 as the lowest abundant of the chosen model biomarkers, we obtained a LOD of 20 pg/mL and an average CV of 6%. Overall, the results suggest that the presented microfluidic device paired with the powerful capabilities of specialized equipment in centralized laboratories could provide a viable solution for remote healthcare applications, opening new possibilities for e-doctor and e-health applications.

## Supporting Information Available

Detailed analytical parameters evaluated for each assay (XLSX)

## References

[ref1] James C. A.; Barfield M. D.; Maass K. F.; Patel S. R.; Anderson M. D. Will Patient-Centric Sampling Become the Norm for Clinical Trials after COVID-19?. Nat. Med. 2020, 26, 1810–1811. 10.1038/s41591-020-01144-1. 33230340

[ref2] Carter L. J.; Garner L. V.; Smoot J. W.; Li Y.; Zhou Q.; Saveson C. J.; Sasso J. M.; Gregg A. C.; Soares D. J.; Beskid T. R.; et al. Assay Techniques and Test Development for COVID-19 Diagnosis. ACS Cent. Sci. 2020, 6, 591–605. 10.1021/acscentsci.0c00501. 32382657 PMC7197457

[ref3] Dakappagari N.; Zhang H.; Stephen L.; Amaravadi L.; Khan M. U. Recommendations for Clinical Biomarker Specimen Preservation and Stability Assessments. Bioanalysis 2017, 9, 643–653. 10.4155/bio-2017-0009. 28508714

[ref4] Kong F. M. S.; Zhao L.; Wang L.; Chen Y.; Hu J.; Fu X.; Bai C.; Wang L.; Lawrence T. S.; Anscher M. S.; et al. Ensuring Sample Quality for Blood Biomarker Studies in Clinical Trials: A Multicenter International Study for Plasma and Serum Sample Preparation. Transl. Lung Cancer Res. 2017, 6, 625–634. 10.21037/tlcr.2017.09.13. 29218266 PMC5709139

[ref5] Sunkara V.; Kumar S.; Sabaté Del Río J.; Kim I.; Cho Y. K. Lab-on-a-Disc for Point-of-Care Infection Diagnostics. Acc. Chem. Res. 2021, 54, 3643–3655. 10.1021/acs.accounts.1c00367. 34516092

[ref6] Johannsen B.; Müller L.; Baumgartner D.; Karkossa L.; Früh S.; Bostanci N.; Karpíšek M.; Zengerle R.; Paust N.; Mitsakakis K. Automated Pre-Analytic Processing of Whole Saliva Using Magnet-Beating for Point-of-Care Protein Biomarker Analysis. Micromachines 2019, 10, 833 10.3390/mi10120833. 31801193 PMC6952956

[ref7] Lin C. T.; Kuo S. H.; Lin P. H.; Chiang P. H.; Lin W. H.; Chang C. H.; Tsou P. H.; Li B. R. Hand-Powered Centrifugal Microfluidic Disc with Magnetic Chitosan Bead-Based ELISA for Antibody Quantitation. Sens. Actuators, B 2020, 316, 1–10. 10.1016/j.snb.2020.128003.

[ref8] Shen M.; Li N.; Lu Y.; Cheng J.; Xu Y. An Enhanced Centrifugation-Assisted Lateral Flow Immunoassay for the Point-of-Care Detection of Protein Biomarkers. Lab Chip 2020, 20, 2626–2634. 10.1039/D0LC00518E. 32567627

[ref9] Lin Q.; Wu J.; Fang X.; Kong J. Washing-Free Centrifugal Microchip Fluorescence Immunoassay for Rapid and Point-of-Care Detection of Protein. Anal. Chim. Acta 2020, 1118, 18–25. 10.1016/j.aca.2020.04.031. 32418600

[ref10] Li F.; You M.; Li S.; Hu J.; Liu C.; Gong Y.; Yang H.; Xu F. Paper-Based Point-of-Care Immunoassays: Recent Advances and Emerging Trends. Biotechnol. Adv. 2020, 107442 10.1016/j.biotechadv.2019.107442. 31470046

[ref11] Suntornsuk W.; Suntornsuk L. Recent Applications of Paper-based Point-of-care Devices for Biomarker Detection. Electrophoresis 2020, 41, 287–305. 10.1002/elps.201900258. 31613392

[ref12] Zhang L.; Du X.; Su Y.; Niu S.; Li Y.; Liang X.; Luo H. Quantitative Assessment of AD Markers Using Naked Eyes: Point-of-Care Testing with Paper-Based Lateral Flow Immunoassay. J. Nanobiotechnol. 2021, 19, 366 10.1186/s12951-021-01111-z. PMC8597216 34789291

[ref13] Vashist S. K. Paper-Based Point-of-Care Immunoassays. In Point-of-Care Technologies Enabling Next-Generation Healthcare Monitoring and Management; Springer International Publishing, 2019; pp 133–155.

[ref14] Joung H. A.; Ballard Z. S.; Wu J.; Tseng D. K.; Teshome H.; Zhang L.; Horn E. J.; Arnaboldi P. M.; Dattwyler R. J.; Garner O. B.; et al. Point-of-Care Serodiagnostic Test for Early-Stage Lyme Disease Using a Multiplexed Paper-Based Immunoassay and Machine Learning. ACS Nano 2020, 14, 229–240. 10.1021/acsnano.9b08151. 31849225

[ref15] Fernandes S. C.; Walz J. A.; Wilson D. J.; Brooks J. C.; Mace C. R. Beyond Wicking: Expanding the Role of Patterned Paper as the Foundation for an Analytical Platform. Anal. Chem. 2017, 89, 5654–5664. 10.1021/acs.analchem.6b03860. 28406607

[ref16] Mattila J.-P.; Amaro A.; Longo M.; Antaki J.; Koirala S.; Gandini A. RapidQ: A Reader-Free Microfluidic Platform for the Quantitation of Antibodies against the SARS-CoV-2 Spike Protein. Biomicrofluidics 2022, 16, 024105 10.1063/5.0079054. 35356130 PMC8933056

[ref17] Dincer C.; Bruch R.; Kling A.; Dittrich P. S.; Urban G. A. Multiplexed Point-of-Care Testing-XPOCT. Trends Biotechnol. 2017, 728–742. 10.1016/j.tibtech.2017.03.013. 28456344 PMC5538621

[ref18] Barbosa A. I.; Reis N. M. A Critical Insight into the Development Pipeline of Microfluidic Immunoassay Devices for the Sensitive Quantitation of Protein Biomarkers at the Point of Care. Analyst 2017, 142, 858–882. 10.1039/C6AN02445A. 28217778

[ref19] Ren A. H.; Diamandis E. P.; Kulasingam V. Uncovering the Depths of the Human Proteome: Antibody-Based Technologies for Ultrasensitive Multiplexed Protein Detection and Quantification. Mol. Cell. Proteomics 2021, 20, 100155 10.1016/j.mcpro.2021.100155. 34597790 PMC9357438

[ref20] Doornekamp L.; Embregts C. W. E.; Aron G. I.; Goeijenbier S.; van de Vijver D. A. M. C.; van Gorp E. C. M.; Geurtsvankessel C. H. Dried Blood Spot Cards: A Reliable Sampling Method to Detect Human Antibodies against Rabies Virus. PLoS Negl. Trop. Dis. 2020, 14, 1–10. 10.1371/journal.pntd.0008784. PMC7584180 33048925

[ref21] Kluge J. A.; Li A. B.; Kahn B. T.; Michaud D. S.; Omenetto F. G.; Kaplan D. L. Silk-Based Blood Stabilization for Diagnostics. Proc. Natl. Acad. Sci. U.S.A. 2016, 113, 5892–5897. 10.1073/pnas.1602493113. 27162330 PMC4889389

[ref22] Evans M. J.; Livesey J. H.; Ellis M. J.; Yandle T. G. Effect of Anticoagulants and Storage Temperatures on Stability of Plasma and Serum Hormones. Clin. Biochem. 2001, 34, 107–112. 10.1016/S0009-9120(01)00196-5. 11311219

[ref23] Chaigneau C.; Cabioch T.; Beaumont K.; Betsou F. Serum Biobank Certification and the Establishment of Quality Controls for Biological Fluids: Examples of Serum Biomarker Stability after Temperature Variation. Clin. Chem. Lab. Med. 2007, 45, 1390–1395. 10.1515/CCLM.2007.160. 17635068

[ref24] Velghe S.; Delahaye L.; Stove C. P. Is the Hematocrit Still an Issue in Quantitative Dried Blood Spot Analysis?. J. Pharm. Biomed. Anal. 2019, 163, 188–196. 10.1016/j.jpba.2018.10.010. 30317075

[ref25] Lawson A. J.; Bernstone L.; Hall S. K. Newborn Screening Blood Spot Analysis in the Uk: Influence of Spot Size, Punch Location and Haematocrit. J. Med. Screen. 2016, 23, 7–16. 10.1177/0969141315593571. 26113437

[ref26] George R. S.; Moat S. J. Effect of Dried Blood Spot Quality on Newborn Screening Analyte Concentrations and Recommendations for Minimum Acceptance Criteria for Sample Analysis. Clin. Chem. 2016, 62, 466–475. 10.1373/clinchem.2015.247668. 26647314

[ref27] Capiau S.; Stove V. V.; Lambert W. E.; Stove C. P. Prediction of the Hematocrit of Dried Blood Spots via Potassium Measurement on a Routine Clinical Chemistry Analyzer. Anal. Chem. 2013, 85, 404–410. 10.1021/ac303014b. 23190205

[ref28] Richardson G.; Marshall D.; Keevil B. G. Prediction of Haematocrit in Dried Blood Spots from the Measurement of Haemoglobin Using Commercially Available Sodium Lauryl Sulphate. Ann. Clin. Biochem. 2018, 55, 363–367. 10.1177/0004563217726809. 28774182

[ref29] Lenk G.; Sandkvist S.; Pohanka A.; Stemme G.; Beck O.; Roxhed N. A Disposable Sampling Device to Collect Volume-Measured DBS Directly from a Fingerprick onto DBS Paper. Bioanalysis 2015, 7, 2085–2094. 10.4155/bio.15.134. 26327187

[ref30] Carling R. S.; Emmett E. C.; Moat S. J. Evaluation of Volumetric Blood Collection Devices for the Measurement of Phenylalanine and Tyrosine to Monitor Patients with Phenylketonuria. Clin. Chim. Acta 2022, 535, 157–166. 10.1016/j.cca.2022.08.005. 35995273

[ref31] Velghe S.; Stove C. P. Evaluation of the Capitainer-B Microfluidic Device as a New Hematocrit-Independent Alternative for Dried Blood Spot Collection. Anal. Chem. 2018, 90, 12893–12899. 10.1021/acs.analchem.8b03512. 30256092

[ref32] Whittaker K.; Mao Y. Q.; Lin Y.; Zhang H.; Zhu S.; Peck H.; Huang R. P. Dried Blood Sample Analysis by Antibody Array across the Total Testing Process. Sci. Rep. 2021, 11, 20549 10.1038/s41598-021-99911-8. 34654894 PMC8521592

[ref33] Hauser J.; Lenk G.; Hansson J.; Beck O.; Stemme G.; Roxhed N. High-Yield Passive Plasma Filtration from Human Finger Prick Blood. Anal. Chem. 2018, 90, 13393–13399. 10.1021/acs.analchem.8b03175. 30379058

[ref34] Hauser J.; Lenk G.; Ullah S.; Beck O.; Stemme G.; Roxhed N. An Autonomous Microfluidic Device for Generating Volume-Defined Dried Plasma Spots. Anal. Chem. 2019, 91, 7125–7130. 10.1021/acs.analchem.9b00204. 31063366

[ref35] Cummins B. M.; Chinthapatla R.; Lenin B.; Ligler F. S.; Walker G. M. Modular Pumps as Programmable Hydraulic Batteries for Microfluidic Devices. Technology 2017, 5, 21–30. 10.1142/S2339547817200011.

[ref36] Häussler R. S.; Bendes A.; Iglesias M. J.; Sanchez-Rivera L.; Dodig-Crnković T.; Byström S.; Fredolini C.; Birgersson E.; Dale M.; Edfors F.; et al. Systematic Development of Sandwich Immunoassays for the Plasma Secretome. Proteomics 2019, 19, 1–19. 10.1002/pmic.201900008. 31278833

[ref37] Drobin K.; Nilsson P.; Schwenk J. M. Highly Multiplexed Antibody Suspension Bead Arrays for Plasma Protein Profiling. Methods Mol. Biol. 2013, 1023, 137–145. 10.1007/978-1-4614-7209-4_8. 23765623

[ref38] Hauser J.; Kylberg G.; Colomb-Delsuc M.; Stemme G.; Sintorn I. M.; Roxhed N. A Microfluidic Device for TEM Sample Preparation. Lab Chip 2020, 20, 4186–4193. 10.1039/D0LC00724B. 33033812

[ref39] Ekins R. P. Multi-Analyte Immunoassay. J. Pharm. Biomed. Anal. 1989, 7, 155–168. 10.1016/0731-7085(89)80079-2. 2488616

[ref40] Said E. A.; Al-Reesi I.; Al-Shizawi N.; Jaju S.; Al-Balushi M. S.; Koh C. Y.; Al-Jabri A. A.; Jeyaseelan L. Defining IL-6 Levels in Healthy Individuals: A Meta-Analysis. J. Med. Virol. 2020, 93, 3915–3924. 10.1002/jmv.26654. 33155686

[ref41] Klevebro S.; Hellgren G.; Hansen-Pupp I.; Wackernagel D.; Hallberg B.; Borg J.; Pivodic A.; Smith L.; Ley D.; Hellström A. Elevated Levels of IL-6 and IGFBP-1 Predict Low Serum IGF-1 Levels during Continuous Infusion of RhIGF-1/RhIGFBP-3 in Extremely Preterm Infants. Growth Horm. IGF Res. 2020, 50, 1–8. 10.1016/j.ghir.2019.11.001. 31756675 PMC7054155

[ref42] Zhang J.; Hao Y.; Ou W.; Ming F.; Liang G.; Qian Y.; Cai Q.; Dong S.; Hu S.; Wang W.; Wei S. Serum Interleukin-6 Is an Indicator for Severity in 901 Patients with SARS-CoV-2 Infection: A Cohort Study. J. Transl. Med. 2020, 18, 406 10.1186/s12967-020-02571-x. 33121497 PMC7594951

[ref43] Vainer N.; Dehlendorff C.; Johansen J. S. Systematic Literature Review of IL-6 as a Biomarker or Treatment Target in Patients with Gastric, Bile Duct, Pancreatic and Colorectal Cancer. Oncotarget 2018, 9, 29820–29841. 10.18632/oncotarget.25661. 30038723 PMC6049875

[ref44] Song J.; Park D. W.; Moon S.; Cho H. J.; Park J. H.; Seok H.; Choi W. S. Diagnostic and Prognostic Value of Interleukin-6, Pentraxin 3, and Procalcitonin Levels among Sepsis and Septic Shock Patients: A Prospective Controlled Study According to the Sepsis-3 Definitions. BMC Infect. Dis. 2019, 19, 968 10.1186/s12879-019-4618-7. 31718563 PMC6852730

[ref45] Posti J. P.; Takala R. S. K.; Lagerstedt L.; Dickens A. M.; Hossain I.; Mohammadian M.; Ala-Seppälä H.; Frantzén J.; van Gils M.; Hutchinson P. J.; et al. Correlation of Blood Biomarkers and Biomarker Panels with Traumatic Findings on Computed Tomography after Traumatic Brain Injury. J. Neurotrauma 2019, 36, 2178–2189. 10.1089/neu.2018.6254. 30760178 PMC6909751

[ref46] Liou L.-b.; Tsai W.; Chang C. J.; Chao W.; Chen M. Blood Monocyte Chemotactic Protein-1 (MCP-1) and Adapted Disease Activity Score28-MCP-1: Favorable Indicators for Rheumatoid Arthritis Activity. PLoS One 2013, 8, 1–9. 10.1371/journal.pone.0055346. PMC3559534 23383162

[ref47] Xu Y. W.; Chen H.; Hong C. Q.; Chu L. Y.; Yang S. H.; Huang L. S.; Guo H.; Chen L. Y.; Liu C. T.; Huang X. Y.; et al. Serum IGFBP-1 as a Potential Biomarker for Diagnosis of Early-Stage Upper Gastrointestinal Tumour. EBioMedicine 2020, 51, 1–9. 10.1016/j.ebiom.2019.11.027. PMC6956950 31901863

[ref48] Ganesan P.; Shanmugam P.; Sattar S. B. A.; Shankar S. L. Evaluation of IL-6, CRP and Hs-CRP as Early Markers of Neonatal Sepsis. J. Clin. Diagnostic Res. 2016, 10, 13–17. 10.7860/JCDR/2016/19214.7764. PMC4948389 27437213

[ref49] Johns I.; Moschonas K. E.; Medina J.; Ossei-Gerning N.; Kassianos G.; Halcox J. P. Risk Classification in Primary Prevention of CVD According to QRISK2 and JBS3 “Heart Age”, and Prevalence of Elevated High-Sensitivity C Reactive Protein in the UK Cohort of the EURIKA Study. Open Heart 2018, 5, e000849 10.1136/openhrt-2018-000849. 30564373 PMC6269641

[ref50] Lee W. J.; Liao Y. C.; Wang Y. F.; Lin I. F.; Wang S. J.; Fuh J. L. Plasma MCP-1 and Cognitive Decline in Patients with Alzheimer’s Disease and Mild Cognitive Impairment: A Two-Year Follow-up Study. Sci. Rep. 2018, 8, 1280 10.1038/s41598-018-19807-y. 29352259 PMC5775300

[ref51] Roxhed N.; Bendes A.; Dale M.; Mattsson C.; Hanke L.; Dodig-Crnković T.; Christian M.; Meineke B.; Elsässer S.; Andréll J.; et al. Multianalyte Serology in Home-Sampled Blood Enables an Unbiased Assessment of the Immune Response against SARS-CoV-2. Nat. Commun. 2021, 12, 3695 10.1038/s41467-021-23893-4. 34140485 PMC8211676

[ref52] Faura J.; Bustamante A.; Reverté S.; García-Berrocoso T.; Millán M.; Castellanos M.; Lara-Rodríguez B.; Zaragoza J.; Ventura O.; Hernández-Pérez M.; et al. Blood Biomarker Panels for the Early Prediction of Stroke-Associated Complications. J. Am. Heart Assoc. 2021, 10, 1–8. 10.1161/JAHA.120.018946. PMC8174272 33634708

[ref53] Ma S.; Wang W.; Xia B.; Zhang S.; Yuan H.; Jiang H.; Meng W.; Zheng X.; Wang X. Multiplexed Serum Biomarkers for the Detection of Lung Cancer. EBioMedicine 2016, 11, 210–218. 10.1016/j.ebiom.2016.08.018. 27575387 PMC5049985

